# Linoleic Acid Attenuates the Toxic Dose of Bupivacaine-Mediated Reduction of Vasodilation Evoked by the Activation of Adenosine Triphosphate-Sensitive Potassium Channels

**DOI:** 10.3390/ijms19071876

**Published:** 2018-06-26

**Authors:** Soo Hee Lee, Dawon Kang, Seong-Ho Ok, Seong-Chun Kwon, Hyun-Jin Kim, Eun-Jin Kim, Jeong-Min Hong, Ji-Yoon Kim, Sung Il Bae, Seungmin An, Ju-Tae Sohn

**Affiliations:** 1 Department of Anesthesiology and Pain Medicine, Gyeongsang National University School of Medicine, Gyeongsang National University Hospital, 15 Jinju-daero 816 beon-gil, Jinju-si 52727, Republic of Korea; lishiuji@naver.com (S.H.L.); mdoksh@naver.com (S.-H.O.); 2 Institute of Health Sciences, Gyeongsang National University, Jinju-si 52727, Republic of Korea; 3 Department of Physiology, Gyeongsang National University School of Medicine, Jinju-si 52727, Republic of Korea; dawon@gnu.ac.kr (D.K.); eunjin@hanmail.net (E.-J.K.); 4 Department of Physiology, Institute of Clinical and Translational Research, Catholic Kwangdong University, College of Medicine, Gangneung 25601, Republic of Korea; skwon2028@cku.ac.kr; 5 Division of Applied Life Sciences (BK21 plus), Gyeongsang National University, 501 Jinju-daero, Jinju 52828, Republic of Korea; hyunjkim@gnu.ac.kr; 6 Department of Food Science & Technology, and Institute of Agriculture and Life Science, Gyeongsang National University, 501 Jinju-daero, Jinju 52828, Republic of Korea; 7 Department of Anesthesia and Pain Medicine, Pusan National University Hospital, Biomed Research Institute, Pusan National University School of Medicine, Busan 49241, Republic of Korea; ccarrot@naver.com; 8 Department of Anesthesiology and Pain Medicine, Gyeongsang National University Hospital, 15 Jinju-daero 816 beon-gil, Jinju-si 52727, Republic of Korea; avadore33@naver.com (J.-Y.K.); snugsoul@naver.com (S.I.B.); beatgg@naver.com (S.A.)

**Keywords:** bupivacaine, K_ATP_ channels, lipid emulsion, linoleic acid, toxic dose, local anesthetic

## Abstract

The goal of this study was to investigate the effect of lipid emulsion on a toxic dose of local anesthetic-mediated reduction of vasodilation evoked by the ATP-sensitive potassium (K_ATP_) channel agonist levcromakalim. The effect of lipid emulsion (LE) and linoleic acid on the local anesthetic-mediated reduction of vasodilation and membrane hyperpolarization evoked by levcromakalim was assessed in isolated endothelium-denuded vessels (rat aorta and mesenteric artery) and aortic vascular smooth muscle cells. The effect of LE and linoleic acid on K_ATP_ channel activity in transfected HEK-293 cells was investigated, as was the effect of LE on bupivacaine concentration. The efficacy of LE in attenuating the local anesthetic-mediated reduction of vasodilation evoked by levcromakalim was correlated with the lipid solubility of the local anesthetic. Linoleic acid attenuated the bupivacaine-mediated reduction of vasodilation evoked by levcromakalim. LE decreased the bupivacaine-mediated reduction of membrane hyperpolarization evoked by levcromakalim but did not significantly alter the mepivacaine-mediated reduction. LE and linoleic acid both reversed the bupivacaine-mediated decrease of K_ATP_ activity and enhanced K_ATP_ activity. LE decreased the bupivacaine concentration. Linoleic acid may be the major contributor to LE-induced attenuation of bupivacaine-mediated reduction of vasodilation evoked by levcromakalim via the direct activation of K_ATP_ channels and indirect effects.

## 1. Introduction

Lipid emulsions effectively treat cardiovascular depression evoked by a toxic dose of local anesthetics [1]. The suggested underlying mechanisms include lipid sink and shuttle, fatty acid supply, enhanced myocardial contractility, attenuation of mitochondrial dysfunction, inhibition of cardiac sodium channel blockade, phosphorylation of glycogen synthase kinase-3β and attenuation of nitric oxide production [1]. A lipid sink, in which a lipid emulsion in the blood efficiently absorbs drugs with high lipid solubility (such as bupivacaine) from organs affected by a toxic dose of the drug, is widely accepted [1]. Toxic doses of bupivacaine cause severe myocardial depression and cardiac electrophysiological alterations, leading to hemodynamic depression [2]. In addition, toxic doses of local anesthetics, including bupivacaine, ropivacaine and mepivacaine, produce vasoconstriction [3,4,5,6]. Taken together with the findings of previous reports, toxic doses of bupivacaine diminish the blood flow to organs as the result of both myocardial depression and increased afterload associated with vasoconstriction [2,3]. Thus, decreased blood flow evoked by the systemic toxicity of local anesthetics produces pathophysiologic states, including ischemia, hypoxia and acidosis, which cause adenosine triphosphate-sensitive potassium (K_ATP_) channel-evoked vasodilation of the vascular smooth muscle as a protective response [7,8]. Unfortunately, local anesthetics such as lidocaine, R-(+)-bupivacaine, and mepivacaine decrease the vasodilation evoked by the K_ATP_ channel agonist levcromakalim in isolated vessels [9,10,11,12]. Furthermore, toxic doses of bupivacaine inhibit the reconstituted K_ATP_ channel activity comprising sulfonylurea receptor (SUR) 2B and inward-rectifying potassium channel (Kir6.1) in the vascular smooth muscle [13]. Among various kinds of lipid emulsions, Intralipid^®^, which is composed of 100% long-chain fatty acids from soybean, is widely used for the treatment of local anesthetic systemic toxicity. The long-chain fatty acids contained in Intralipid^®^ are composed of linoleic (53%), oleic (24%), palmitic (11%), alpha-linoleic (8%) and stearic acids (4%) [14]. Moreover, linoleic acid, which comprises the largest group (53%) of long-chain fatty acids in Intralipid^®^, activates K_ATP_ channels and causes membrane hyperpolarization in pancreatic β-cells [15]. Various types of K_ATP_ channels are expressed in multiple vascular smooth muscle cells [7,8]. The K_ATP_ channels have key roles in regulating the resting membrane potential of smooth muscle cells and in the vascular responses to a variety of endogenous and pharmacological vasodilators [7,8]. The control of K_ATP_ channels contributes to the physiological regulation of vascular tone and blood flow [7,8]. However, the effect of lipid emulsions and linoleic acid on the local anesthetic-mediated reduction of the vasodilation evoked by K_ATP_ channels in isolated vessels remains unknown. Based on these previous reports, we tested the hypothesis that lipid emulsions and linoleic acid reverse the toxic dose of local anesthetic-mediated reduction of vasodilation evoked by levcromakalim [1,14,15]. The objective of this study was to examine the effect of a lipid emulsion and linoleic acid on the toxic dose of local anesthetic-mediated reduction of levcromakalim-evoked vasodilation in isolated vessels, including rat aorta and mesenteric artery, and to examine the underlying mechanism.

## 2. Results

**Figure 1:**
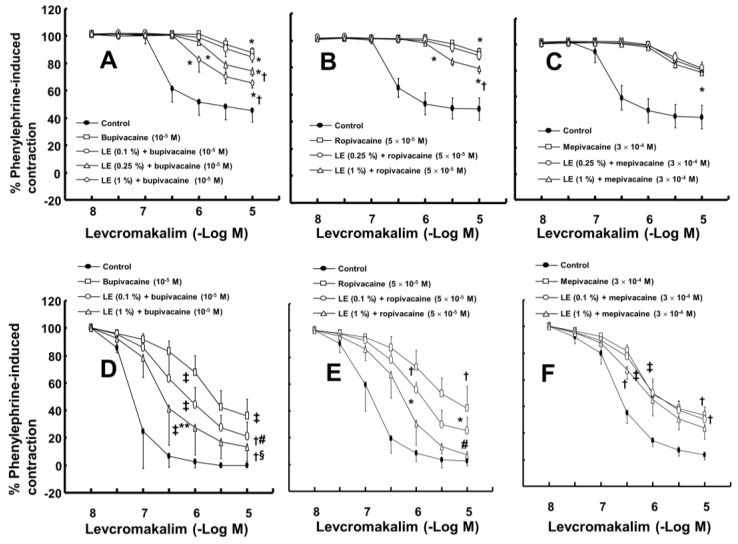
(**A**–**C**): Effect of lipid emulsion (LE) on the toxic dose of local anesthetic (bupivacaine: (**A**), *n* = 10; ropivacaine: (**B**), *n* = 10; mepivacaine: (**C**), *n* = 10)-mediated reduction of levcromakalim-evoked vasodilation in isolated rat aortas without endothelium. Data are expressed as the mean ± standard deviation and shown as a percentage of the maximal contraction evoked by phenylephrine. Data were analyzed by a one-way analysis of variance (ANOVA) followed by Bonferroni’s multiple comparison test, unpaired Student’s *t*-test or a Kruskal-Wallis test with Dunn’s multiple comparison test. *n* indicates the number of isolated rat aortas. Maximal vasodilation induced by levcromakalim (10^−5^ M) or the logarithm (log ED_50_) of the levcromakalim concentration needed to produce the half maximal vasodilation induced by levcromakalim (10^−5^ M): * *p <* 0.001 versus control, † *p* < 0.001 versus bupivacaine or ropivacaine alone. (**D**–**F**): Effect of LE on the toxic dose of local anesthetic-mediated reduction of levcromakalim-evoked vasodilation in isolated rat mesenteric arteries without endothelium. Data (bupivacaine: (**D**), *n* = 7; ropivacaine: (**E**), [control *n* = 6, ropivacaine *n* = 5, 0.1% LE + ropivacaine: *n* = 6 and 1% LE + ropivacaine: *n* = 5]; mepivacaine: (**F**), *n* = 7) are expressed as the mean ± SD and shown as a percentage of the maximal contraction evoked by phenylephrine. Data were analyzed by one-way ANOVA followed by Bonferroni’s multiple comparison test or a Kruskal-Wallis test with Dunn’s multiple comparison test. *n* indicates the number of isolated rat mesenteric arteries. Maximal vasodilation induced by levcromakalim (10^−5^ M) or log ED_50_: * *p* < 0.05, † *p* < 0.01 and ‡ *p* < 0.001 versus control. # *p* < 0.05, § *p* < 0.01 and ** *p* < 0.001 versus bupivacaine or ropivacaine alone.

**Figure 2:**
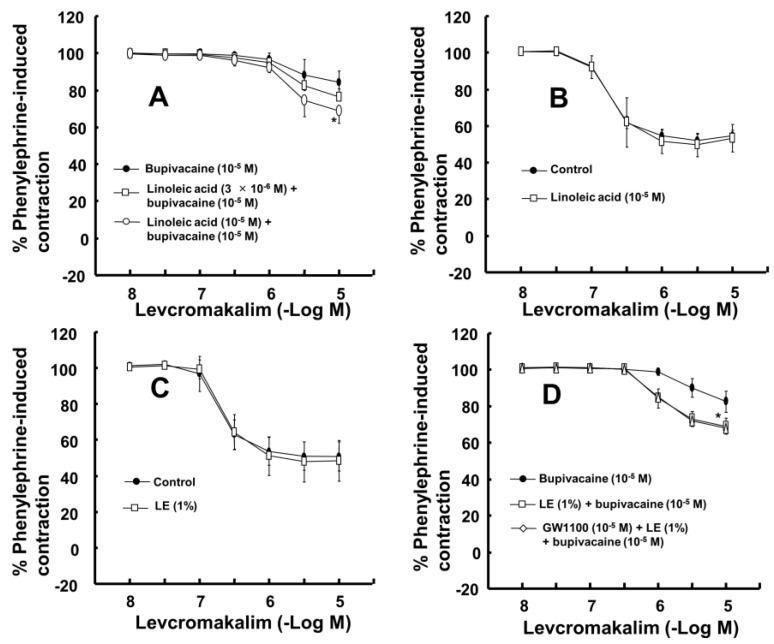
(**A**): Effect of linoleic acid on the toxic dose of bupivacaine-mediated reduction of levcromakalim-evoked vasodilation in isolated rat aortas without endothelium. Data (*n* = 9) are expressed as the mean ± SD and shown as a percentage of the contraction evoked by phenylephrine. Data were analyzed by a Kruskal-Wallis test with Dunn’s multiple comparison test. *n* indicates the number of isolated rat aortas. Maximal vasodilation induced by levcromakalim (10^−5^ M): * *p* < 0.001 versus bupivacaine. (**B**): Effect of linoleic acid alone on the levcromakalim-evoked vasodilation in isolated rat aortas without endothelium. Data (*n* = 8) are expressed as the mean ± SD and shown as a percentage of the contraction evoked by phenylephrine. *n* indicates the number of isolated rat aortas. (**C**,**D**): Effect of lipid emulsion (LE) ((**C**), *n* = 10) alone on the levcromakalim-evoked vasodilation in isolated rat aortas without endothelium and the effects of LE ((**D**), *n* = 10) alone and combined treatment (*n* = 10) with GW1100 and LE on the toxic dose of bupivacaine-mediated reduction of levcromakalim-evoked vasodilation in isolated rat aortas without endothelium. Data are expressed as the mean ± SD and shown as a percentage of the contraction evoked by phenylephrine. Data were analyzed by one-way ANOVA followed by Bonferroni’s multiple comparison test or unpaired Student’s *t*-test. *n* indicates the number of isolated rat aortas. Maximal vasodilation induced by levcromakalim (10^−5^ M): * *p* < 0.001 versus bupivacaine alone.

**Figure 3:**
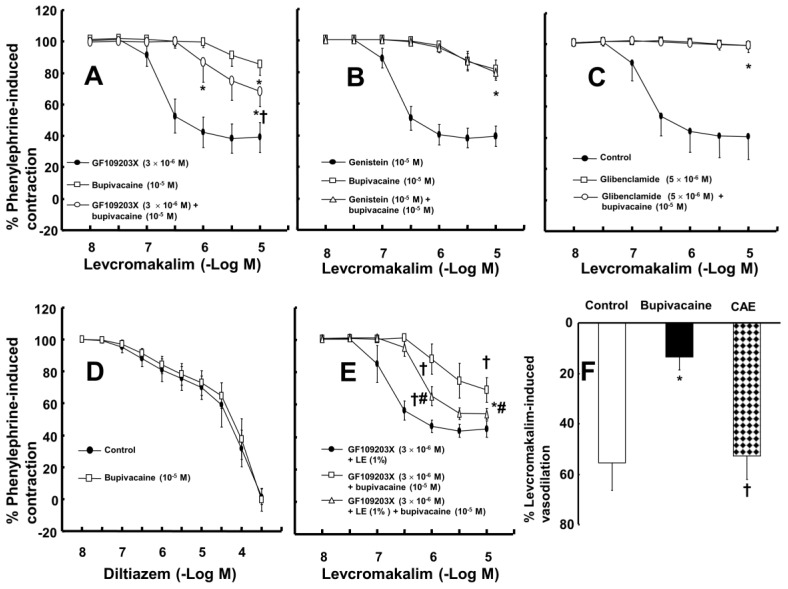
(**A**,**B**): Effect of GF109203X ((**A**), *n* = 10) and genistein ((**B**), *n* = 10) on the toxic dose of bupivacaine-mediated reduction of levcromakalim-evoked vasodilation in isolated rat aortas without endothelium. Data are expressed as the mean ± SD and shown as a percentage of the contraction evoked by phenylephrine. Data were analyzed by one-way analysis of variance (ANOVA) followed by Bonferroni’s multiple comparison test or Mann-Whitney test. *n* indicates the number of isolated rat aortas. Maximal vasodilation induced by levcromakalim (10^−5^ M) or the logarithm (log ED_50_) of the levcromakalim concentration needed to produce half of the maximal vasodilation induced by levcromakalim (10^−5^ M): * *p* < 0.001 versus GF109023X or genistein alone, † *p* < 0.001 versus bupivacaine alone. (**C**,**D**): Effect of glibenclamide ((**C**), *n* = 10) on the toxic dose of bupivacaine-mediated reduction of levcromakalim-evoked vasodilation in isolated rat aortas without endothelium and the effect of bupivacaine ((**D**), *n* = 10) on the diltiazem-evoked vasodilation in isolated rat aortas without endothelium. Data are expressed as the mean ± SD and shown as a percentage of the contraction evoked by phenylephrine. Data were analyzed by one-way ANOVA followed by Bonferroni’s multiple comparison test or unpaired Student’s *t*-test. *n* indicates the number of isolated rat aortas. Maximal vasodilation induced by levcromakalim (10^−5^ M): * *p* < 0.001 versus control. (**E**): Effect of GF109203X and the combined treatment of GF109203X and lipid emulsion (LE) on the toxic dose of bupivacaine-mediated reduction of levcromakalim-evoked vasodilation in isolated rat aortas without endothelium. Data (*n* = 10) are expressed as the mean ± SD and shown as a percentage of the contraction evoked by phenylephrine. Data were analyzed by one-way ANOVA followed by Bonferroni’s multiple comparison test. *n* indicates the number of isolated rat aortas. Maximal vasodilation induced by levcromakalim (10^−5^ M) or log ED_50_: * *p* < 0.01 and † *p* < 0.001 versus GF109203X plus LE, # *p* < 0.001 versus GF109203X plus bupivacaine. (**F**): Effect of bupivacaine (10^−5^ M) alone and centrifuged aqueous extract (CAE), which corresponds to the bupivacaine (10^−5^ M)-containing LE (1%) obtained from a mixture of LE and bupivacaine by ultracentrifugation, on the levcromakalim (10^−5^ M)-induced vasodilation from the phenylephrine-induced contraction of rat aortas without endothelium. Data (*n* = 8) are expressed as the mean ± SD and shown as a vasodilation percentage from the maximal contraction evoked by phenylephrine. Data were analyzed by one-way ANOVA followed by Bonferroni’s multiple comparison test. *n* indicates the number of isolated rat aortas. * *p* < 0.001 versus control. † *p* < 0.001 versus bupivacaine alone.

The magnitude of the contraction evoked by phenylephrine before adding levcromakalim or diltiazem to the organ bath was not significantly different among all groups, including the control and drug-treated groups, in each experimental protocol. A toxic dose of bupivacaine (10^−5^ M), ropivacaine (5 × 10^−5^ M) and mepivacaine (3 × 10^−4^ M) decreased vasodilation evoked by levcromakalim in the isolated endothelium-denuded rat aorta and mesenteric artery (Figure 1A–F). Lipid emulsion (0.25% and 1%) significantly attenuated the toxic dose of bupivacaine-mediated reduction of maximal vasodilation evoked by levcromakalim (10^−5^ M) in isolated endothelium-denuded rat aorta (Figure 1A, *p* < 0.001), and the highest concentration of lipid emulsion (1%) only significantly attenuated the ropivacaine-mediated reduction of maximal vasodilation evoked by levcromakalim (10^−5^ M) (Figure 1B, *p* < 0.001). Lipid emulsion (0.1% and 1%) significantly inhibited the bupivacaine-mediated reduction of maximal vasodilation evoked by levcromakalim (10^−5^ M) in isolated rat mesenteric artery (Figure 1D; *p* < 0.05), and lipid emulsion (1%) significantly attenuated the bupivacaine-mediated increase in log ED_50_ evoked by levcromakalim (Figure 1D; log ED_50_: *p <* 0.001). The highest concentration of lipid emulsion (1%) used in this experiment only significantly attenuated the ropivacaine-mediated reduction of maximal vasodilation evoked by levcromakalim (10^−5^ M) in isolated endothelium-denuded mesenteric artery (Figure 1E; *p* < 0.05). However, lipid emulsion did not significantly alter the toxic dose of mepivacaine-mediated reduction of vasodilation evoked by levcromakalim in the isolated rat aorta and mesenteric artery (Figure 1C,F). Linoleic acid (10^−5^ M) significantly attenuated the toxic dose of bupivacaine-mediated reduction of maximal vasodilation evoked by levcromakalim (10^−5^ M) (Figure 2A; *p* < 0.001 versus bupivacaine alone). However, the linoleic acid (10^−5^ M) and lipid emulsion (1%) had no effect on the vasodilation evoked by levcromakalim (Figure 2B,C). In addition, the free fatty acid receptor antagonist GW1100 (10^−5^ M) had no effect on the lipid emulsion reversal of the bupivacaine-mediated reduction of vasodilation evoked by levcromakalim (Figure 2D). GF109203X (3 × 10^−6^ M) significantly attenuated the bupivacaine (10^−5^ M)-mediated reduction of levcromakalim (10^−5^ M)-evoked maximal vasodilation (Figure 3A; *p* < 0.001 versus bupivacaine alone). However, genistein (10^−5^ M) had no effect on the bupivacaine (10^−5^ M)-mediated reduction of vasodilation evoked by levcromakalim (Figure 3B). In addition, bupivacaine had no effect on the levcromakalim-evoked vasodilation in isolated endothelium-denuded rat aorta pretreated with glibenclamide (5 × 10^−6^ M) (Figure 3C). Bupivacaine did not significantly change the diltiazem-evoked vasodilation (Figure 3D). The combined pretreatment with GF109203X and lipid emulsion resulted in a significantly greater reversal of the bupivacaine-mediated reduction of levcromakalim-evoked vasodilation than pretreatment with GF109203X alone (Figure 3E; log ED_50_ and maximal vasodilation: *p* < 0.01). Levcromakalim (10^−5^ M)-evoked vasodilation was significantly stronger in the Krebs solution containing centrifuged aqueous extract (CAE, corresponding to a mixture of 1% lipid emulsion and 10^−5^ M bupivacaine) than the Krebs solution containing 10^−5^ M bupivacaine alone (Figure 3F; *p* < 0.001).

**Figure 4:**
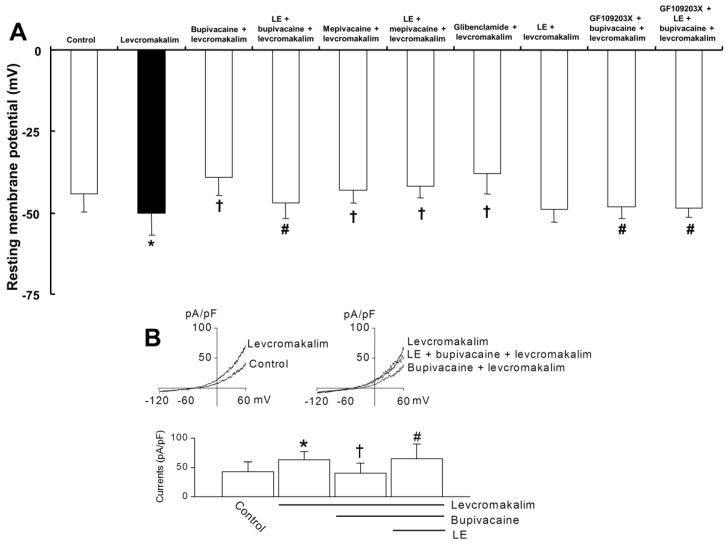
Effect of bupivacaine and lipid emulsion (LE) on membrane potential and whole-cell currents of levcromakalim-treated vascular smooth muscle cells. (**A**): Effects of bupivacaine (10^−5^ M), mepivacaine (3 × 10^−4^ M), LE (1%), glibenclamide (5 × 10^−6^ M) and GF109203X (3 × 10^−6^ M), alone or combined, on the membrane potential induced by levcromakalim (10^−5^ M) in rat aortic vascular smooth muscle cells. Membrane potentials were recorded under current-clamp mode (*I* = 0). Data (*n* = 9) are expressed as the mean ± SD. Data were analyzed using one-way analysis of variance (ANOVA) followed by post hoc comparisons using Tukey’s test. *n* indicates the number of independent experiments. * *p* < 0.05 versus control. † *p* < 0.05 versus levcromakalim alone. # *p* < 0.05 versus bupivacaine + levcromakalim. (**B**): Whole-cell currents recorded in smooth muscle cells. The currents were activated by levcromakalim and were blocked by bupivacaine. LE quenched the effect of bupivacaine. The bar graph summarizes the effects of levcromakalim, bupivacaine and LE on the whole-cell currents. The current levels were determined at +60 mV. Data (*n* = 6) are expressed as the mean ± SD. Data were analyzed using one-way ANOVA followed by post hoc comparisons using Tukey’s test. * *p* < 0.05 versus control. † *p* < 0.05 versus levcromakalim alone. # *p* < 0.05 versus bupivacaine + levcromakalim.

Lipid emulsion (1%) significantly reversed the bupivacaine (10^−5^ M)-mediated reduction of membrane hyperpolarization evoked by levcromakalim (10^−5^ M) (Figure 4A; *p* < 0.05 versus bupivacaine plus levcromakalim). However, the lipid emulsion did not significantly alter the mepivacaine (3 × 10^−4^ M)-mediated reduction of membrane hyperpolarization evoked by levcromakalim (Figure 4A; *p* = 0.42). Glibenclamide (5 × 10^−6^ M) significantly attenuated the membrane hyperpolarization evoked by levcromakalim (Figure 4A; *p =* 0.001), whereas the lipid emulsion (1%) did not significantly alter the membrane hyperpolarization evoked by levcromakalim (Figure 4A; *p* = 0.51). GF109203X alone or GF109203X plus lipid emulsion significantly reversed the bupivacaine (10^−5^ M)-mediated reduction of membrane hyperpolarization evoked by levcromakalim (Figure 4A; *p* < 0.05 versus bupivacaine plus levcromakalim). In vascular smooth muscle cells, the whole-cell currents evoked by levcromakalim (10^−5^ M) were significantly inhibited by bupivacaine treatment (Figure 4B; *p <* 0.05 versus levcromakalim). The effect of bupivacaine was significantly blocked by 1% lipid emulsion (Figure 4B; *p <* 0.05 versus bupivacaine + levcromakalim).

**Figure 5:**
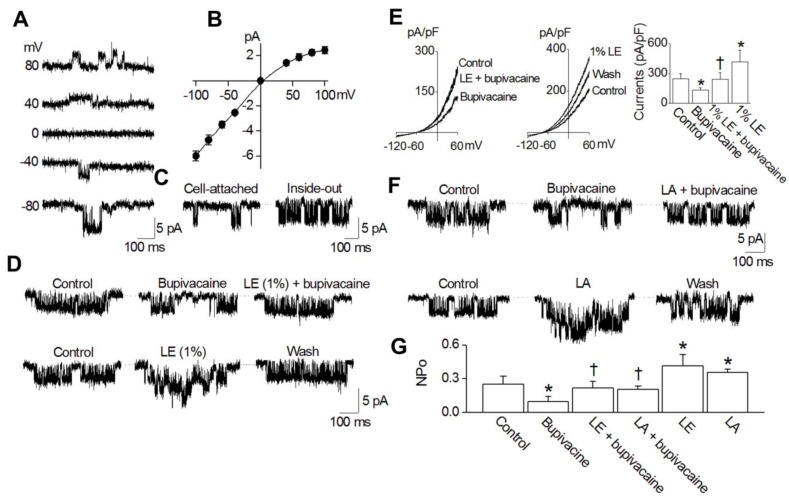
Direct effect of bupivacaine, lipid emulsion (LE) and linoleic acid (LA) on K_ATP_ channels. (**A**): Current tracings typically observed from a cell-attached patch of HEK-293 cells transfected with rat Kir6.2 and SUR2A show single-channel openings at various membrane potentials in symmetrical 150 mM KCl conditions. (**B**): A current-voltage relationship of K_ATP_ channels in 150 mM KCl is shown. Each point is the mean of five determinations. (**C**): Opening of K_ATP_ channels in inside-out patches. A cell-attached patch was formed at −80 mV; then, the inside-out patch was formed. (**D**): Blocking of the bupivacaine-induced inhibition of K_ATP_ channel activity by LE. In the inside-out patch, bupivacaine application (10^−4^ M) to the cytosolic side of the membrane inhibited channel activity, and the combined treatment with LE (1%) and bupivacaine (10^−4^ M) recovered the bupivacaine-induced inhibition of channel activity. LE alone activated K_ATP_ channels, and the LE effect was reversible. (**E**): Effect of bupivacaine and LE on whole-cell currents of K_ATP_ channels. Whole-cell currents showing the effect of bupivacaine and LE. A bar graph showing the effects of bupivacaine and LE on K_ATP_ channels. (**F**): Blocking of the bupivacaine-induced inhibition of K_ATP_ channel activity by LA (2 × 10^−5^ M). Currents were recorded as in (**D**) except for LA treatment. (**G**): Summary of K_ATP_ channel activity by bupivacaine, LE, and LA alone and combination treatment. Data are expressed as the mean ± SD (*n* = 5). Data were analyzed using one-way analysis of variance (ANOVA) followed by post hoc comparisons using Tukey’s test. *n* indicates the number of independent experiments. * *p* < 0.05 versus control. † *p* < 0.05 versus 10^−4^ M bupivacaine alone. The currents measured at +80 mV of pipette potential were analyzed.

To confirm that bupivacaine and lipid emulsion modulate K_ATP_ channels directly, we recorded single-channel currents in HEK-293 cells transfected with Kir6.2 and SUR2 in a 150 mM KCl bath solution. Single-channel recording showed that the K_ATP_ channels were active at all membrane potentials tested (−80 mV to +80 mV). The openings and closings of several channels in a typical cell-attached patch are shown in Figure 5A. The current-voltage relationship obtained from the amplitude obtained at each membrane potential was slightly inwardly rectifying in symmetrical 150 mM KCl conditions (Figure 5B). At a membrane potential of +80 and −80 mV, the single-channel conductances were 28-pS and 59-pS, respectively. When inside-out patches were formed, the K_ATP_ channel opening was strong (Figure 5C). Under inside-out patch mode, at −80 mV, bupivacaine and lipid emulsion were applied to the bath solution. Bupivacaine (10^−4^ M) attenuated channel activity in the HEK-293 cells transfected with Kir6.2 and SUR2 (Figure 5D). However, lipid emulsion (1%) reversed the bupivacaine (10^−4^ M)-mediated reduction of channel activity (Figure 5D). In addition, lipid emulsion (1%) alone enhanced channel activity (Figure 5D). In the whole-cell mode, lipid emulsion blocked the bupivacaine-induced inhibition of K_ATP_ currents (Figure 5E). Lipid emulsion alone increased the currents by 1.6-fold, and the lipid emulsion effect was reversible (Figure 5E). In addition to lipid emulsion, linoleic acid (2 × 10^−5^ M) reversed the bupivacaine (10^−4^ M)-mediated reduction of K_ATP_ channel activity (Figure 5F). Furthermore, linoleic acid (2 × 10^−5^ M) alone enhanced K_ATP_ channel activity (Figure 5F). Figure 5G summarizes the K_ATP_ activity (NPo) in response to bupivacaine, lipid emulsion and linoleic acid, alone and combined.

**Figure 6:**
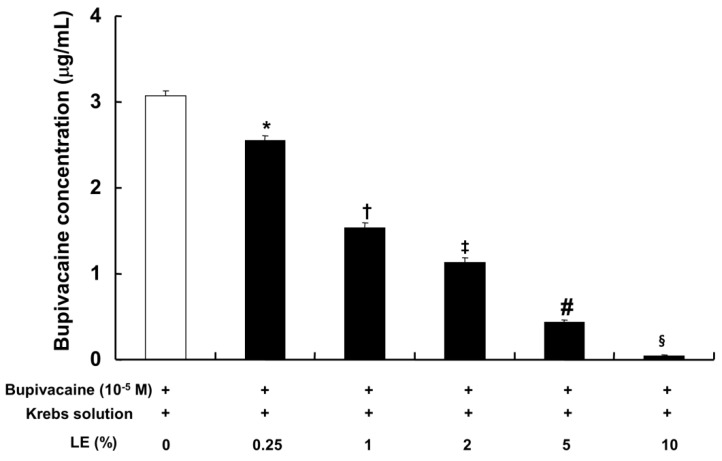
Effect of lipid emulsion (LE) on bupivacaine (10^−5^ M) in Krebs solution. After removal of the emulsified bupivacaine and lipid, the remaining bupivacaine in the centrifuged aqueous extract was measured by ultraperformance liquid chromatography-quadrupole time-of-flight mass spectrometry in the multiple reaction monitoring mode. Data (*n* = 5) are expressed as the mean ± SD. Data were analyzed by one-way analysis of variance (ANOVA) with Duncan’s test using SPSS 17.0 (SPSS Inc., Chicago, IL, USA). *n* indicates the number of independent experiments. * *p* < 0.001 versus bupivacaine alone, † *p* < 0.001 versus bupivacaine + LE (0.25%), ‡ *p* < 0.001 versus bupivacaine + LE (1%), # *p <* 0.001 versus bupivacaine + LE (2%), **§** *p <* 0.001 versus bupivacaine + LE (5%).

The CAE (0.25% to 10%), which was obtained from the lipid emulsion (0.25% to 10%) and bupivacaine (10^−5^ M) in Krebs solution by centrifugation, exhibited a significantly lower bupivacaine concentration in a concentration-dependent manner compared with bupivacaine (10^−5^ M) in Krebs solution without lipid emulsion (Figure 6; *p* < 0.001).

**Figure 7:**
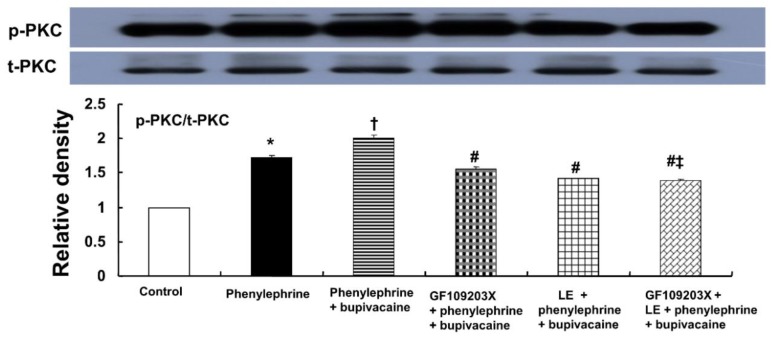
Effects of bupivacaine (10^−5^ M), GF109203X (3 × 10^−6^ M) and lipid emulsion (LE, 1%), alone or combined, on the protein kinase C (PKC) phosphorylation evoked by phenylephrine (10^−6^ M) in rat aortic vascular smooth muscle cells (RAVSMCs). RAVSMCs were treated with phenylephrine (10^−6^ M) alone for 10 min, pretreated with phenylephrine (10^−6^ M) for 1 min followed by bupivacaine (10^−5^ M) for 9 min, pretreated with 3 × 10^−6^ M GF109203X for 70 min followed by 10^−6^ M phenylephrine for 1 min and subsequently treated with 10^−5^ M bupivacaine for 9 min, pretreated with LE (1%) for 40 min followed by phenylephrine (10^−6^ M) treatment for 1 min and subsequently treated with 10^−5^ M bupivacaine for 9 min, or pretreated with GF109203X for 30 min followed by LE (1%) for 40 min and subsequently treated with phenylephrine (10^−6^ M) for 1 min and post-treated with bupivacaine (10^−5^ M) for 9 min. PKC phosphorylation was examined by Western blot analysis as described in the Materials and Methods. Data (*n* = 3) are expressed as the mean ± SD. Data were analyzed using one-way analysis of variance (ANOVA) followed by Bonferroni’s multiple comparison test. *n* indicates the number of independent experiments. p-PKC: phosphorylated PKC. t-PKC: total PKC. * *p* < 0.001 versus control. † *p* < 0.001 versus phenylephrine alone. # *p* < 0.001 versus phenylephrine + bupivacaine. ‡ *p* < 0.001 versus GF109203X + phenylephrine + bupivacaine.

Bupivacaine (10^−5^ M) significantly enhanced the PKC phosphorylation evoked by phenylephrine (10^−6^ M) (Figure 7; *p* < 0.001 versus phenylephrine alone). Pretreatment with GF109203X (3 × 10^−6^ M) or lipid emulsion (1%) significantly attenuated the bupivacaine (10^−5^ M)-mediated increase in PKC phosphorylation evoked by phenylephrine (Figure 7; *p* < 0.001 versus phenylephrine plus bupivacaine). The combined pretreatment with GF109203X and lipid emulsion exhibited a significantly greater inhibition of the bupivacaine-mediated increase in PKC phosphorylation evoked by phenylephrine than pretreatment with GF109203X alone (Figure 7; *p* < 0.001).

## 3. Discussion

This study is the first to show that lipid emulsion attenuates the toxic dose of bupivacaine-mediated reduction of vasodilation evoked by K_ATP_ channels via indirect and direct action. The indirect action appears to be associated with the sequestration of highly lipid-soluble bupivacaine by lipid emulsion (Intralipid^®^) containing linoleic acid as the largest portion of its long-chain fatty acids.

Similar to a previous report using isolated rat mesenteric artery, levcromakalim (10^−5^ M) in the current study produced almost complete vasodilation of phenylephrine-induced contractions in isolated rat mesenteric artery [16]. In agreement with the lipid sink theory, lipid emulsion reversed the bupivacaine-induced blockade of the fast sodium current to a greater extent than it reversed the mepivacaine-induced blockade of the fast sodium current in cardiomyocytes [17]. The increasing order of magnitude of reduction in the local anesthetic concentration due to the sequestration of local anesthetics induced by lipid emulsion is reportedly as follows: Mepivacaine, ropivacaine, and bupivacaine [18,19]. Furthermore, lipid emulsion treatment after local anesthetic-induced cardiac arrest decreases recovery time in cardiac arrest induced by the most lipid-soluble local anesthetic, bupivacaine, but has no effect on the recovery time from cardiac arrest induced by the least lipid-soluble local anesthetic, mepivacaine [20]. The increasing order of lipid solubility of these local anesthetics is as follows: mepivacaine, lidocaine, ropivacaine and bupivacaine [21]. Given the local anesthetic’s lipid solubility, the ability of the lipid emulsion to reverse the toxic dose of local anesthetic-mediated reduction of vasodilation and hyperpolarization evoked by levcromakalim in the present study was correlated with the local anesthetic’s lipid solubility, supporting the lipid sink theory, which is consistent with previous reports [1,17,18,19,20,21]. In the mesenteric artery, which is regarded as a small resistance arteriole contributing to organ blood flow regulation, the potency of reversal evoked by lipid emulsion was also associated with lipid solubility (Figure 1D–F) [22]. Moreover, lipid emulsion blocked the bupivacaine-mediated decrease in whole-cell currents evoked by levcromakalim (Figure 4B). Additionally, the CAE, which was obtained from Krebs solution containing lipid emulsion and bupivacaine by centrifugation, reduced bupivacaine concentration in a concentration-dependent manner (Figure 6). The CAE obtained from the mixture of lipid emulsion and bupivacaine reportedly attenuated the blood pressure decrease evoked by bupivacaine in an in vivo guinea pig model and the sodium channel inhibition evoked by bupivacaine in cardiomyocytes [17,23]. Consistent with previous reports, ultracentrifuged aqueous extract (corresponding to the mixture of 1% lipid emulsion and 10^−5^ M bupivacaine) nearly abolished the toxic dose of bupivacaine (10^−5^ M)-mediated reduction of vasodilation evoked by levcromakalim (Figure 3F), suggesting that the upper lipid layer remaining after ultracentrifugation resulted in the absorption of a larger amount of bupivacaine, leading to a decrease in the bupivacaine concentration to below the threshold concentration needed to block levcromakalim-induced vasodilation [17,23]. In addition, under inside-out patch mode, lipid emulsion (1%) both reversed bupivacaine (10^−4^ M)-mediated inhibition of K_ATP_ channel activity and enhanced K_ATP_ channel activity without bupivacaine (Figure 5D,G). Taken together, these results suggest that lipid emulsion has a direct and indirect effect on K_ATP_ channels. The free fatty acid receptor activated by fatty acid is involved in various cellular responses [24]. However, as the free fatty acid receptor antagonist GW1100 had no effect on the lipid emulsion-induced reversal of the toxic dose of bupivacaine-mediated reduction of vasodilation evoked by levcromakalim (Figure 2D), this result suggests that reversal evoked by lipid emulsion did not involve activation of the free fatty acid receptor.

The largest group (53%) of long-chain fatty acids of Intralipid^®^, a recommended lipid emulsion for the treatment of local anesthetic-evoked systemic toxicity by the American Society of Regional Anesthesia and Pain Medicine, is the polyunsaturated fatty acid linoleic acid (C18:2n-6) [14,25]. In this study, a dose of linoleic acid (10^−5^ M), which was lower than the approximately calculated concentration of linoleic acid (1.9 × 10^−2^ M) contained in Intralipid^®^ (1%), reversed the toxic dose of bupivacaine-mediated reduction of vasodilation evoked by levcromakalim (Figure 2A). In addition, linoleic acid (2 × 10^−5^ M) reversed the toxic dose of bupivacaine (10^−4^ M)-mediated reduction of K_ATP_ channel activity (Figure 5F,G). Furthermore, similar to previous reports that linoleic acid stimulates K_ATP_ channels in pancreatic ß-cells and insulinoma cells, both linoleic acid (2 × 10^−5^ M) and lipid emulsion (1%), which intrinsically includes approximately 1.9 × 10^−2^ M linoleic acid, enhanced K_ATP_ channel activity in the absence of bupivacaine (Figure 5D,F,G) [15,26]. Thus, linoleic acid appears to be the major long-chain fatty acid that mediates the lipid emulsion-induced reversal of the bupivacaine-mediated reduction of K_ATP_ channel activity via direct and indirect action. In addition, the linolenic and stearic acid contained in Intralipid^®^ attenuate the human sodium channel in HEK-293 cells but reverse the bupivacaine-induced blockade of the sodium channel in HEK-293 cells, suggesting that the fatty acids contained in Intralipid^®^ may have a modulatory effect on the sodium channel blockade by bupivacaine [27]. The current results supporting the presumed indirect mechanism associated with linoleic acid-mediated sequestration of bupivacaine are as follows: Linoleic acid (10^−5^ M) had no effect on levcromakalim-induced vasodilation. However, the CAE obtained from the mixture of lipid emulsion (1%), with 1.9 × 10^−2^ M linoleic acid, and bupivacaine (10^−5^ M) decreased bupivacaine concentration to 49% ± 2% (Figure 6). Thus, further study regarding the effect of linoleic acid on bupivacaine concentration is needed. Lipid emulsion (1%) and linoleic acid (10^−5^ M) had no effect on the levcromakalim-induced vasodilation (Figure 2B,C), whereas lipid emulsion (1%) or linoleic acid (2 × 10^−5^ M) alone increased K_ATP_ channel activity (Figure 5G). This discrepancy may be due to the differences in experimental method (tension study versus electrophysiological study), species (aortic rings versus transfected HEK-293 cells) and the chemical interaction of various long-chain fatty acids in Intralipid^®^. As lipid emulsions other than Intralipid^®^ effectively treat drug toxicity, the type of lipid emulsion and fatty acid that efficiently recovers the K_ATP_ channel blockade evoked by toxic doses of local anesthetics remains to be determined [28,29,30].

PKC or tyrosine kinase was associated with the modulation of the K_ATP_ channel activity in vascular smooth muscle [31,32,33]. The PKC-mediated pathway contributes to the vasoconstriction evoked by levobupivacaine, which comprises S-enantiomers of bupivacaine [34]. Several vasoconstrictors block K_ATP_ channels via PKC activation in vascular smooth muscle [35,36,37]. GF109203X reversed the bupivacaine-mediated reduction of vasodilation evoked by levcromakalim, whereas genistein had no effect on the bupivacaine-mediated reduction (Figure 3A,B). These results suggest that bupivacaine-mediated reduction of vasodilation evoked by levcromakalim is mediated by PKC activation. Consistent with the above finding, the PKC inhibitor GF109203X reversed the bupivacaine-mediated reduction of membrane hyperpolarization evoked by levcromakalim (Figure 4A) and attenuated the bupivacaine-mediated enhancement of PKC phosphorylation evoked by phenylephrine (Figure 7). As 1% lipid emulsion had no effect on levcromakalim-induced vasodilation and did not significantly affect membrane hyperpolarization induced by levcromakalim, this increased reversal of bupivacaine-evoked inhibition by the combined treatment with GF109203X and lipid emulsion (Figure 3E) may be ascribed to an additional lipid-emulsion-mediated sequestration of bupivacaine. Glibenclamide nearly abolished bupivacaine-mediated reduction of vasodilation evoked by levcromakalim and inhibited hyperpolarization evoked by levcromakalim, suggesting that bupivacaine blocks the regulatory SUR of K_ATP_ channels [7]. However, as bupivacaine did not significantly affect the diltiazem-induced vasodilation, the bupivacaine-mediated reduction of vasodilation evoked by levcromakalim may be specific. The combined pretreatment with GF109203X and lipid emulsion decreased bupivacaine-mediated enhancement of PKC phosphorylation evoked by phenylephrine compared with GF109203X alone (Figure 7), whereas there was no significant difference in membrane hyperpolarization evoked by levcromakalim between the combined treatment with GF109203X plus lipid emulsion and GF109203X alone (Figure 4A). This discrepancy may be due to differences in experimental methods, such as the Western blot used to support the tension study using phenylephrine and the electrophysiological study used to examine the membrane potential changes induced by levcromakalim.

Taken together, these results suggest that lipid emulsion treatment may reverse the bupivacaine-induced inhibition of the protective vasodilation evoked by K_ATP_ channels that are activated during hypoxia, acidosis and ischemia; this ischemia is due to the decreased blood flow induced by local anesthetic systemic toxicity [1,7,8]. However, clinical extrapolation of the results of this study has several limitations. First, local anesthetic-evoked enhanced endothelial nitric oxide release and lipid-emulsion-evoked reduction of nitric oxide release may contribute to the enhancement and decrease in vasodilation evoked by levcromakalim, respectively, which may be confounding factors in this study [3,4,6,38,39]. Thus, l-NAME-pretreated endothelial denuded vessels were used. Second, the activation of K_ATP_ channels evoked pathophysiologically by cardiovascular collapse during systemic toxicity of local anesthetics may be different from that of K_ATP_ channels evoked by the K_ATP_ channel agonist levcromakalim [7,8]. Third, this experiment did not consider other factors; for example, the heart and autonomic nervous system are involved in hemodynamic control in vivo. Fourth, lipid emulsion pretreatment was used in this study, whereas lipid emulsion is used as a clinical post-treatment after cases of cardiovascular collapse caused by a toxic dose of local anesthetics.

## 4. Materials and Methods

All experimental methods and protocols (GNU-160414-R0019, 6th April, 2016) were approved by Gyeongsang National University and Catholic Kwandong University. All experimental protocols were performed in accordance with the Regulation for the Care and Use of Laboratory Animals stipulated by Gyeongsang National University and Catholic Kwandong University.

### 4.1. Preparation of Isolated Rat Aorta and Mesenteric Artery and Isometric Tension Measurements

Male Sprague-Dawley rats (body weight: 250–300 g, 95 total rats) in small cages were anesthetized using 100% carbon dioxide inhalation supplied through a small tube attached to the cage. Preparation for isometric tension measurement of the rat aorta was performed as described previously [9]. The descending thoracic aorta was dissected and isolated from the thoracic cavity. Then, perivascular connective tissue of the isolated rat aorta immersed in Krebs solution was removed under a microscope. The components of Krebs solution are as follows: 118 mM NaCl, 4.7 mM KCl, 1.2 mM MgSO_4_, 2.4 mM CaCl_2_, 25 mM NaHCO_3_ and 11 mM glucose. The isolated aorta was cut into rings with a 2.5 mm length. The endothelium was removed from the isolated descending thoracic aorta by inserting 25-gauge needles into the lumen of the isolated aorta and rolling the isolated aorta for a few seconds. The isolated rat aorta was suspended on a Grass isometric transducer (FT-03; Grass Instrument, Quincy, MA, USA) connected to a 10 mL organ bath maintained at 37 °C. The 3.0 g resting tension was used for isolated rat aorta and maintained for 120 min to reach equilibrium [9,34,40]. The Krebs solution in the organ bath was aerated with oxygen (95%) and carbon dioxide (5%) to maintain the pH from 7.35 to 7.45. The Krebs solution contained in the organ bath with the suspended isolated rat aorta was replaced by fresh Krebs solution every 30 min. After the phenylephrine (10^−8^ M)-induced contraction reached a plateau, acetylcholine (10^−5^ M) was added to the organ bath to verify endothelial removal. A lower than 15% relaxation evoked by acetylcholine was considered endothelial denudation in the isolated rat aorta. Then, the isolated rat aorta was washed several times with fresh Krebs solution to restore baseline resting tension.

Preparation for isometric tension measurement of the rat mesenteric artery was performed as described previously [41]. The mesenteric artery was quickly excised and placed in cold Krebs solution. The vessels were cut into 1-mm-wide ring segments and were placed in 3 mL tissue baths on two L-shaped hooks, one of which was attached to a force transducer (MLT050, ADInstruments, Colorado Springs, CO, USA) for isometric measurement of tension. The tension of the vessel was recorded on a computer equipped with PowerLab/400 (ADInstruments). The data were analyzed by the Chart 5 program. The 0.5 g resting tension was used for the isolated rat mesenteric artery and maintained for 90 min to research equilibrium before each experiment [42]. The endothelium was mechanically removed by gentle rubbing using moistened cotton, and its absence was verified by the lack of an acetylcholine-induced relaxant response (10^−6^ M) of the contraction evoked by 10^−5^ M phenylephrine. The isolated rat mesenteric artery was then washed with fresh Krebs solution to restore baseline resting tension.

The following experimental protocols were performed using endothelium-denuded aortic and mesenteric arterial rings with nitric oxide synthase inhibitor N^W^-nitro-l-arginine methyl ester (l-NAME, 10^−4^ M) treatment. All isolated vessels were used only one time to produce concentration-response curves evoked by the vasorelaxant (levcromakalim or diltiazem). Isolated vessels obtained from same animal were used to obtain the dose-response curves evoked by levcromakalim or diltiazem in the presence or absence of pretreatment drugs in each experimental protocol. The rationale to use l-NAME-pretreated endothelium-denuded vessels in this experiment is as follows: First, nitric oxide that is basally produced from the endothelium produces more levcromakalim-induced vasodilation in endothelium-intact rat aorta than endothelium-denuded rat aorta via the cyclic guanosine monophosphate pathway [8,43]. In addition, the direct effect of local anesthetics on the isolated rat aorta causes vasoconstriction, which is attenuated by endothelial nitric oxide production stimulated by local anesthetics [3,4,6]. Thus, we used endothelium-denuded vessels to avoid compounding factors, such as enhanced levcromakalim-induced vasodilation caused by increased endothelial nitric oxide release mediated by the local anesthetic itself. Second, as residual endothelium remaining after endothelial denudation may affect levcromakalim-induced vasodilation, endothelial denuded vessels were pretreated with L-NAME in this experiment [9].

### 4.2. Experimental Protocols

First, the effect of lipid emulsion (Intralipid^®^ 20%) on the toxic dose of local anesthetic-mediated alteration of vasodilation evoked by levcromakalim was examined in isolated endothelium-denuded rat aorta and mesenteric artery pretreated with l-NAME. Some of the isolated vessels were treated with lipid emulsion (0.1%, 0.25% and 1%) for 20 min before adding phenylephrine to the organ bath. When contraction evoked by 10^−6^ and 10^−5^ M phenylephrine was developing in the isolated rat aorta and mesenteric artery, respectively, isolated vessels were treated with a toxic dose of local anesthetics (10^−5^ M bupivacaine, 5 × 10^−5^ M ropivacaine and 3 × 10^−4^ M mepivacaine). Based on previous reports of the plasma concentration of local anesthetics causing systemic toxicity, anesthetic potency and a pilot study to examine relative potency of the local anesthetic-mediated reduction of vasodilation evoked by levcromakalim, toxic concentrations of local aesthetics used in this experiment were chosen [44,45,46]. We added the local anesthetic to the organ bath after the initiation of contraction by phenylephrine because vasoconstriction evoked by bupivacaine, ropivacaine and mepivacaine slightly enhances baseline resting tension [3,4,5,6]. After contraction evoked by phenylephrine reached a sustained level in the presence or absence of local anesthetic alone or lipid emulsion plus local anesthetic, levcromakalim (10^−8^ to 10^−5^ M) was added to the organ bath to produce cumulative levcromakalim dose-response curves.

Second, both the effect of linoleic acid on the toxic dose of bupivacaine-mediated reduction of vasodilation evoked by levcromakalim and the effect of linoleic acid or lipid emulsion alone on the vasodilation evoked by levcromakalim were examined in isolated endothelium-denuded rat aorta with L-NAME treatment. Aortic rings were treated with linoleic acid (3 × 10^−6^ and 10^−5^ M) or lipid emulsion (1%) for 20 min before adding phenylephrine to the organ bath. When contraction evoked by phenylephrine (10^−6^ M) was developing, the aortic rings were treated with 10^−5^ M bupivacaine. After phenylephrine produced a sustained and stable contraction in the presence or absence of linoleic acid, lipid emulsion and bupivacaine alone and combined treatment with linoleic acid plus bupivacaine, levcromakalim was added to the organ bath to produce cumulative levcromakalim dose-response curves. In addition, the effect of the free fatty acid receptor antagonist GW1100 on lipid emulsion reversal of the toxic dose of bupivacaine-mediated reduction of vasodilation evoked by levcromakalim was investigated in isolated endothelium-denuded rat aorta with L-NAME treatment. Aortic rings were pretreated with GW1100 (10^−5^ M) for 20 min followed by post-treatment with lipid emulsion (1%) for 20 min or pretreated with lipid emulsion (1%) alone for 20 min before adding phenylephrine to the organ bath [47]. When contraction evoked by phenylephrine (10^−6^ M) was developing, all aortic rings were post-treated with 10^−5^ M bupivacaine in the presence or absence of lipid emulsion alone or combined treatment with GW1100 plus lipid emulsion. After phenylephrine produced a sustained and stable contraction, levcromakalim (10^−8^ to 10^−5^ M) was added to produce the cumulative levcromakalim dose-response curve.

Third, the effects of the protein kinase C (PKC) inhibitor GF109203X (3 × 10^−6^ M), the tyrosine kinase inhibitor genistein (10^−5^ M) and the K_ATP_ channel antagonist glibenclamide (5 × 10^−6^ M) on the toxic dose of bupivacaine-mediated reduction of levcromakalim-evoked vasodilation were examined in isolated endothelium-denuded rat aortas with l-NAME treatment. Aortic rings were treated with each inhibitor for 20 min before adding phenylephrine to the organ bath. When contraction evoked by phenylephrine was developing, aortic rings were treated with 10^−5^ M bupivacaine. After phenylephrine (10^−6^ M) produced a sustained and stable contraction in the presence or absence of each inhibitor or bupivacaine alone and combined treatment with each inhibitor plus bupivacaine, levcromakalim was added to the organ bath to produce cumulative levcromakalim dose-response curves. In addition, the effect of bupivacaine on the vasodilation induced by the calcium channel blocker diltiazem was examined in isolated endothelium-denuded rat aorta with l-NAME treatment. When contraction evoked by phenylephrine (10^−6^ M) was developing, aortic rings were treated with 10^−5^ M bupivacaine. After phenylephrine produced a stable and sustained contraction in the presence or absence of bupivacaine, diltiazem (10^−8^ to 3 × 10^−4^ M) was added to produce cumulative diltiazem dose-response curves.

Fourth, we compared the effect of GF109203X alone and combined treatment with GF109203X and lipid emulsion on the bupivacaine-mediated reduction of vasodilation evoked by levcromakalim in isolated endothelium-denuded rat aorta with L-NAME treatment. Aortic rings were treated with GF109203X (3 × 10^−6^ M) alone for 40 min or pretreated with GF109203X (3 × 10^−6^ M) for 20 min, followed by post-treatment with lipid emulsion (1%) for 20 min before adding phenylephrine to the organ bath. When contraction evoked by phenylephrine (10^−6^ M) was developing in the presence of GF109203X alone or combined treatment with GF109203X and lipid emulsion, some of aortic rings were treated with 10^−5^ M bupivacaine. After phenylephrine (10^−6^ M) produced a stable and sustained contraction, levcromakalim (10^−8^ to 10^−5^ M) was added to produce cumulative dose-response curves.

Finally, for confirmation that sequestration of bupivacaine by lipid emulsion would contribute to lipid emulsion reversal of the bupivacaine-mediated reduction of vasodilation evoked by levcromakalim, the effect of CAE without the upper lipid layer, which was obtained from a mixture of lipid emulsion and bupivacaine by ultracentrifugation (75,000 *g* for 18 min at 4 °C) using an Optima^TM^ HAX-XP Ultracentrifuge (Beckman Counter, Brea, CA, USA), and bupivacaine alone on the vasodilation evoked by levcromakalim (10^−5^ M) was examined in isolated endothelium-denuded rat aorta with l-NAME treatment. When contraction evoked by phenylephrine was developing, aortic rings were treated with bupivacaine (10^−5^ M) alone or CAE corresponding to a mixture of 1% lipid emulsion and 10^−5^ M bupivacaine. After phenylephrine (10^−6^ M) produced a sustained and stable contraction, levcromakalim (10^−5^ M) was added to the organ bath to investigate levcromakalim (10^−5^ M)-evoked vasodilation in isolated endothelium-denuded rat aorta with bupivacaine (10^−5^ M) treatment alone or CAE corresponding to a mixture of 1% lipid emulsion and 10^−5^ M bupivacaine.

### 4.3. Cell Culture

Vascular smooth muscle cells isolated from rat thoracic aortas by enzymatic digestion were cultured in Dulbecco’s modified Eagle’s medium (Carlsbad, CA, USA) supplemented with 10% heat-inactivated fetal bovine serum, 100 U/mL of penicillin, and 100 mg/mL of streptomycin as described previously in our laboratory [9]. Cells were grown at 37 °C in 5% CO_2_, and the medium was changed every 2 days until the cells reached confluence. Cells were propagated through trypsin treatment. Cells at passage number 5~9 were seeded into 100-mm dishes at a density of 10^7^ cells and incubated until the cells reached 70% confluence. Confluent cells were further incubated in serum-free medium for 15 h before the experiments. For electrophysiological studies, vascular smooth muscle cells and HEK-293 cells were plated on poly-l-lysine-coated glass coverslips in a culture dish and incubated at 37 °C in 5% CO_2_. The vascular smooth muscle cells and HEK-293 cells were used 1 day after plating and 2–3 days after transfection, respectively.

### 4.4. Electrophysiological Studies

Electrophysiological recording was performed in rat aortic vascular smooth muscle cells and transfected HEK-293 cells using a patch clamp amplifier (Axopatch 200, Axon Instruments, Union City, CA, USA) as described previously [48]. Briefly, single-channel currents were recorded in HEK-293 cells transfected with DNA fragments encoding rat Kir6.2 (AB043638), ATP-binding cassette subfamily C member 9 (Abcc9 [SUR2], NM_013040) and green fluorescent protein in pcDNA3.1. The currents were filtered at 2 kHz using an 8-pole Bessel filter (−3 dB; Frequency Devices, Haverhill, MA, USA) and transferred to a computer (Samsung) using the Digidata 1320 interface (Axon Instruments, Union City, CA, USA) at a sampling rate of 20 kHz. Threshold detection of channel openings was set at 50%. Single-channel currents were analyzed with the pCLAMP program (version 10, Axon) with filter dead time of 100 µs (0.3/cutoff frequency). Channel activity (NPo, where N is the number of channels in the patch and Po is the probability of a channel being open) was determined from ~1–2 min of current recording. The single-channel current tracings shown in the figures were filtered at 2 kHz. In experiments using cell-attached and excised patches, the pipette and bath solutions contained the following (mM): 150 KCl, 1 MgCl_2_, 5 EGTA and 10 HEPES (pH 7.3). The pH was adjusted to the desired values with HCl or KOH. For whole-cell currents, bath solution contained the following (mM): 135 NaCl, 5 KCl, 1 CaCl_2_, 1 MgCl_2_, 5 glucose, and 10 HEPES, and pipette solution contained the following (mM): 150 KCl, 1 MgCl_2_, 5 EGTA, and 10 HEPES (pH 7.3). All solutions were prepared with Milli-Q water (18.2 MΩ-cm at 25 °C). The whole-cell current was recorded in response to a voltage ramp (−120 to +60 mV; 865 ms duration) from a holding potential of −80 mV. All experiments were performed at ~25 °C. The data were analyzed using Clampfit (pCLAMP, version 9.2, Axon Instruments) and Origin^®^ (version 6.0, Microcal Software, MA, USA).

Membrane potential was measured in rat aortic cultured vascular smooth muscle cells using the whole-cell patch clamp technique under current-clamp mode (*I* = 0), as described previously [9].

### 4.5. Effect of Lipid Emulsion on Bupivacaine Concentration in the Krebs Solution 

Bupivacaine (10^−5^ M) dissolved in the Krebs solution was mixed with Intralipid^®^ at different concentrations (0%, 0.25%, 1%, 2%, 5%, and 10%) by a rotator for 30 min to emulsify the bupivacaine and lipid. After centrifugation at 75,000 *g* for 40 min, the unemulsified bupivacaine in the aqueous layer was measured by ultraperformance liquid chromatography-quadrupole time-of-flight mass spectrometry (UPLC-Q-TOF MS; Waters, Milford, MA, USA). The unemulsified bupivacaine samples were injected into an Acquity UPLC BEH C_18_ column (100 × 2.1 mm, 1.7 µm; Waters) equilibrated with water containing 0.1% formic acid and eluted with a linear acetonitrile containing a 0.1% formic acid gradient (5–100%) at a flow rate of 0.35 mL/min for 7 min. The eluted bupivacaine was analyzed by Q-TOF MS (Waters) using the multiple reaction monitoring and positive electrospray ionization modes. The capillary and sampling cone voltages were set at 3 kV and 30 V, respectively. The desolvation temperature and flow rate were 100 °C and 800 L/h, respectively, and the source temperature was set at 400 °C. Lock spray with leucine-enkephalin (556.2771 Da) was used at a frequency of 10 s to ensure reproducibility and accuracy for all analyses. For quantitative analysis of bupivacaine, a multiple reaction monitoring mode was used, and the precursor and product ions for bupivacaine were 289.21 and 140.13, respectively. All mass data were collected and analyzed by UIFI version 1.8.2 (Waters).

### 4.6. Western Blot

Western blot analysis was performed using the method described by Baik et al. [9]. Cells grown in 100-mm dishes were treated with drugs, including phenylephrine, and washed twice with ice-cold phosphate-buffered saline. For immunoblot analysis, cells were lysed in RIPA lysis buffer (Cell Signaling Technology, Beverly, MA, USA) to obtain total cell lysates. Cell lysates were centrifuged to remove debris at 14,000 rpm for 15 min at 4 °C, and the resulting supernatants (30 µg of protein) were separated by sodium dodecyl sulfate-polyacrylamide gel electrophoresis on a 10% gel for 90 min at 110 V. The separated proteins were transferred to polyvinylidene difluoride membranes using Trans-Blot^®^ SD Semi-Dry Transfer Cells (Bio-Rad Laboratories, Hercules, CA, USA). After the membranes were blocked in 5% bovine serum albumin (BioShop, Burlington, ON, Canada) in Tris-buffed saline (pH 7.0), they were incubated overnight at 4 °C with primary antibodies (anti-PKC and anti-phospho-PKC) at a dilution of 1:1000 in 5% bovine serum albumin in Tris-buffed saline containing Tween-20. Immune complexes were detected with SuperSignal^®^ West Pico chemiluminescent substrate (Thermo Scientific, Rockford, IL, USA). Band density was determined by densitometric analysis software (Bio-Rad, Hercules, CA, USA).

### 4.7. Drugs

All drugs are commercially available and of the highest purity. Levcromakalim was obtained from Tocris Bioscience (Bristol, United Kingdom). GF109203X, glibenclamide, linoleic acid, diltiazem, L-NAME, phenylephrine and acetylcholine were purchased from Sigma-Aldrich (St. Louis, MO, USA). GW1100 was purchased from Calbiochem (San Diego, CA, USA). Intralipid^®^ was donated by Fresenius Kabi Korea (Seoul, Korea). Bupivacaine and mepivacaine were obtained from Reyon Pharmaceutical Co., Ltd. (Seoul, Korea) and Hana Pharmaceutical Co., Ltd. (Gyeonggi-do, Korea), respectively. Ropivacaine was donated by Korea AstraZeneca (North Ryde, NSW, Australia). Anti-phospho-PKC antibody and anti-PKC antibody were obtained from Cell Signaling Technology (Beverly, MA, USA) and Santa Cruz Biotechnology (Santa Cruz, CA, USA), respectively. GF109203X, GW1100 and glibenclamide were dissolved in dimethyl sulfoxide (final dimethyl sulfoxide concentration: less than 0.1%). Levcromakalim was dissolved in ethanol (final ethanol concentration: 0.19%). Molar concentration and percentage were used to express the final organ bath concentration of the drug and lipid emulsion, respectively. Unless stated otherwise, drugs were dissolved in distilled water and subsequently diluted in distilled water.

### 4.8. Statistical Analysis

All the data are expressed as the mean ± SD. Vasodilation evoked by levcromakalim and diltiazem is expressed as the percentage of the maximal contraction evoked by phenylephrine. Normality was assessed using Shapiro-Wilk tests for all data obtained from the isometric tension study. The logarithm (log ED_50_) of drug concentration to produce 50% of the maximal vasodilation evoked by levcromakalim (10^−5^ M) or diltiazem was calculated using nonlinear regression analysis by fitting the dose-response curves evoked by levcromakalim or diltiazem to sigmoidal curves of Prism 5.0 (GraphPad Software, San Diego, CA, USA). When the maximal vasodilation induced by levcromakalim (10^−5^ M) in the presence of the pretreatment drug was less than approximately 25%, comparison among groups was performed using only the maximal vasodilation evoked by levcromakalim (10^−5^ M) because the maximal vasodilation was small and the dose-response curves evoked by levcromakalim did not fit well into the sigmoidal dose-response curve. The effects of several pretreatment drugs on the contraction evoked by phenylephrine before addition of levcromakalim or diltiazem to the organ bath were analyzed using one-way ANOVA followed by Bonferroni’s multiple comparison test or unpaired Student’s *t*-test. *p* values less than 0.05 were considered statistically significant.

## 5. Conclusions

In conclusion, these results suggest that lipid emulsion, specifically linoleic acid, attenuates the bupivacaine-mediated reduction of vasodilation that is evoked by the activation of K_ATP_ channels, which is associated with the high lipid solubility of bupivacaine. Linoleic acid seems to be the major contributor to the lipid emulsion-induced attenuation of the bupivacaine-mediated reduction of the vasodilation evoked by the activation of K_ATP_ channels via both the direct activation of K_ATP_ channels and indirect effects.

## Abbreviations

K_ATP_ channel	ATP-sensitive potassium channel
l-NAME	N^W^-nitro-l-arginine methyl ester
Kir	inward-rectifying potassium channel
SUR	sulfonylurea receptor
log ED_50_	logarithm of drug concentration to produce 50% of the maximal vasodilation
PKC	protein kinase C
CAE	Centrifuged aqueous extract
